# Regeneration of acinar cells following ligation of rat submandibular gland retraces the embryonic-perinatal pathway of cytodifferentiation

**DOI:** 10.1016/j.diff.2009.11.005

**Published:** 2010-02

**Authors:** Emanuele Cotroneo, Gordon B. Proctor, Guy H. Carpenter

**Affiliations:** Salivary Research Unit, floor 17 Tower Wing, King's College London Dental Institute, London,UK, SE1 9RT.

**Keywords:** Salivary gland, Regeneration, SMG-B, PSP, Self-proliferation, Cell precursors

## Abstract

Rat submandibular gland can regenerate following ligation-induced atrophy, eventually recovering its normal morphology and function. Previous studies have suggested that the regeneration process implies both self-proliferation of existing acini and formation of new acinar cells. One hypothesis is that new acinar cells may differentiate from the ductal cells in a similar fashion to the process of cytodifferentiation occurring during submandibular glandular development. In this study atrophy was induced, under recovery anaesthesia, by applying a metal clip on the main duct of the submandibular gland without including the chorda lingual nerve. After 2 weeks the duct was deligated for 3, 5 or 7 days or 8 weeks and the glands collected. Tissue was prepared for immunohistochemstry, biochemical analysis and RNA extraction. The histology of the regenerated glands shows several normal-looking acini, which have regained their glycoprotein content (AB/PAS positive), data also confirmed by biochemical analysis (SDS-PAGE/PAS). Regenerating tissue was characterized by the presence of embryonic-like branched structures ending with AB/PAS positive acinar cells. The proteins SMG-B and PSP are normally expressed in acinar cell precursors during development but only by intercalated ductal cells in the adult stage. In the adult regenerating gland mRNA levels of both SMG-B and PSP were found to be up-regulated compared to ligated glands and SMG-B expression localized to acinar cells whilst the ductal cells were negative. This study of rat submandibular gland regeneration suggests new acinar cells have differentiated from ducts and express markers of acinar cell precursors in a similar manner to the cytodifferentiation process occurring during glandular development.

## 1 Introduction

Salivary gland development represents one of the most typical exemplum of epithelial–mesenchyme interaction (Pispa and Thesleff, 2003). It begins with the invagination of a terminal bud from the oral epithelium into the surrounding mesenchyme which later provides the stimulus for the formation of a branched epithelial tree (Cutler and Chaudhry, 1973). This process known as branching morphogenesis, gives arise to the ductal system and marks the start of the cytodifferentiation, which in the rat submandibular gland carries on postnatally (Cutler and Chaudhry, 1974; Patel et al., 2006; Ball et al., 1991).

The main secretory cell type apparent at birth is not the same as in adult. In fact, the perinatal cells fall into two transient secretory cell types: Type I (or terminal tubules cells), and Type III (or pro-acinar cells). The latter cell type will eventually differentiate into mature acinar cells (Cutler and Chaudhry, 1974; Ball and Redman, 1984). Type III cells are characterized by the expression of specific perinatal proteins known as SMG-A (23.5 kDa, also known as parotid specific protein; PSP), SMG-B1 (26 kDa), and B2 (27.5 kDa) (Ball et al., 1988; Man et al., 1995), which are secreted in response to beta-adrenergic stimulation since the early stage (Ball et al., 1988). SMG-A is encoded by the *psp* gene whilst SMG-B1 and B2 are differentially glycosylated forms of the *smg-b* gene (Mirels et al., 1998). During the perinatal stage of development Type III cells also start to express adult acini markers (i.e. glutamine/glutammatic acid-rich proteins and mucin) and ultimately differentiate into mature acinar cells (Denny et al., 1997; Moreira et al., 1991).

By the adult stage SMG B1 and 2 proteins are no longer present in the acini but are expressed in some of the intercalated duct cells (Ball et al., 1988; Man et al., 1995; Cotroneo et al., 2008). In contrast SMG-A (also known as PSP) does not seem to be expressed in the adult stage (Mirels et al., 1998).

With prolonged duct ligation adult submandibular gland undergoes atrophy leading to secretory dysfunction (Martinez et al., 1982; Osailan et al., 2006a, 2006b; Shiba et al., 1972; Tamarin, 1971a, 1971b, 1971c). The impaired functionality has been linked to the loss of acinar cells due to apoptosis (Takahashi et al., 2000, 2007). Although single clip duct ligation may not be as effective as ligatures it has been shown that some shrunken acini are still present in the atrophic tissue created by either method but they are thought to be in a quiescent, non-functional state (Cotroneo et al., 2008; Tamarin, 1971a, 1971b, 1971c). On the other hand, during early atrophy the ductal cells actively proliferate (Takahashi et al., 2000). When the ligation is removed the submandibular gland is able to recover its functionality regenerating the secretory tissue through both proliferation of residual acini and differentiation of new acinar cells (Takahashi et al., 2004a, 2004b). In a recent study on the early regenerated submandibular gland (3 day of deligation) we suggested that newly formed acini differentiate from unique branched structures present in this tissue. Furthermore in the same study, we have identified immature acini exhibiting a perinatal-like immunoreactive phenotype as they expressed the perinatal protein SMG-B (Cotroneo et al., 2008).

Therefore, the aim of this paper has been to follow the differentiation of the new acinar cells during the progression of submandibular gland regeneration following duct ligation-induced atrophy, and to establish any correlation with the embryonic stage of development based on both morphological and molecular evidence. The characterization of this process should provide useful information for future studies aiming to restore the glandular functionality.

## 2 Materials and methods

### 2.1 Experimental procedure

Twenty five Wistar strain (250–350 g) rats were used. All experimental procedures were conducted with the approval of the local ethics committee and Home Office license. Rats were divided into five groups (*n*=5 each): one group of unoperated controls, a second group experienced 2 weeks of duct ligation. The third, fourth and fifth groups underwent 2 weeks ligation followed by deligation after 3, 5, 7 days or 8 weeks, respectively. Contralateral glands were not used as controls in this study as previously noted compensatory hyperplasia occurred when the other gland was extirpated (Shwartz-Arad et al., 1991), or ligated (Walker and Gobe, 1987). All animals were killed by an anaesthetic overdose.

Atrophy was induced following the intraoral duct ligation procedure as previously described (Osailan et al., 2006a, 2006b). Under recovery anaesthesia (combined Ketamine 75 mg/kg and Xylazine 15 mg/kg intraperitoneally [i.p.]) a metal clip was applied to the main excretory ducts of the submandibular and sublingual glands through a small incision in the floor of the mouth, which was then sutured. A small plastic tube was applied together with the clip in order to avoid fibrosis of the ducts (Osailan et al., 2006a, 2006b). Ligation performed in this manner did not include the chorda lingual nerve. Prior to the collection of the glands following the ligation-only procedure, the presence of the clip and the tube on the duct was confirmed in each animal. After 2 weeks the glands of the ligation-only group were removed under terminal anaesthesia (pentobarbitone 60 mg/kg i.p) and weighed. In the other groups (ligation followed by deligation), the duct was de-ligated after 2 weeks (under recovery anaesthesia), and the glands were collected after a further 3, 5, 7 days or 8 weeks. The collection of the glands was performed under terminal anaesthesia (pentobarbitone 60 mg/kg i.p.). After removal the submandibular gland was carefully dissected from the sublingual gland and weighed. A third of the gland was fixed in formol sucrose for histological sections; another third was snap-frozen (in liquid nitrogen) for the purpose of RNA extraction or for later homogenization and the rest was incubated in cell culture media with added collagenase for cell preparation (see below).

### 2.2 Histochemical staining of tissue sections

Tissue fixed in formol sucrose was embedded in wax and 5 μm sections were cut and mounted on slides. For general morphology tissue sections were then stained with Mayer's Haematoxylin and 1% Eosin (H&E). The secretory granules inside the acinar cells were identified by Alcian Blue periodic acid Schiff's (AB/PAS) staining.

### 2.3 Immunohistochemistry on tissue sections

The tissue sections were first de-waxed and then incubated in a solution of 3% hydrogen peroxide to inhibit the endogenous peroxidase. After being washed in phosphate-buffered saline (PBS; 0.1 M), the sections were incubated with normal goat serum (DAKO, Ely UK, 1:5 dilution of PBS) to avoid non-specific binding of the primary antibody. To investigate the presence of both SMG-B isoforms, tissue sections were incubated with rabbit anti-ZZ3 delta polyclonal antibody (Mirels et al., 1998) (kind gift of Prof Mirels, and Dr Hand) in a 1:50 dilution of PBS. The secondary antibody was biotinylated goat anti-rabbit polyclonal (DAKO, Ely UK, 1:400 dilution); so sections were reacted with streptavidin–biotin horse-radish peroxidase complex (DAKO, Ely UK). The peroxidase activity was visualized with diaminobenzidine tetrahydrochloride (DAB) (0.5 mg/ml) and counterstained with Mayer’s Haematoxylin. A similar method was used for Ki-67 (1:50 dilution of rabbit polyclonal anti-Ki-67, Thermo Scientific, Runcorn, Cheshire, UK) and smooth muscle actin staining (DAKO clone 1A4, Ely UK, 1:50 dilution). Sections for all antibodies were incubated at 95 °C for 15 min in citric acid buffer (pH 6.0) before being incubated with the goat serum. The Ki-67 positive nuclei were counted to assess cell proliferation in the experimental tissues.

### 2.4 Cell preparation

A third of the gland was minced and digested with collagenase (0.5 mg/ml Worthington Biochemical Corporation, Freehold, N.J. USA) as previously described (Xu et al., 1996). The tissue was incubated at 37 °C for 45 min in presence of 100% oxygen; then centrifuged (1000*g*) and cells re-suspended in 10 ml of new media without collagenase and incubated in presence of oxygen at 37 °C for another 30 min.

### 2.5 Immunocytochemistry with Confocal Laser Microscopy

After incubation in buffer without collagenase the cells were washed with PBS and then fixed in 100% methanol for 10 min at room temperature. After washing in PBS they were sequentially incubated in blocking buffers at a different concentration of BSA (0.1% and 1% w/v). Then cells were incubated with goat anti-AQP5 monoclonal antibody (Santa Cruz, CA USA, 1:50 dilution). The negative controls were incubated with 1% BSA blocking buffer and secondary antibody only. After washing with PBS the secondary fluorescent antibodies (anti-goat Alexa 568, Invitrogen, Paisley UK,) were applied on the cells in 1:2000 dilution. Finally the cells were washed, and mounted on slides with MOWIOL solution.

Using a Leica TCS SP2 confocal microscope optical sections (1 μm) were captured at various depths, projected as a three-dimensional (3D) image and colour-coded according to the depth of field.

### 2.6 Tissue homogenization: PAS staining & Western blotting

Snap frozen gland tissue was homogenized in the following buffer solution: TRIS 50 mM, 0.15% TRITON X-100 plus a protease inhibitor cocktail (1:10 dilution, CALBIOCHEM, USA), centrifuged for 15 min, then the supernatant was collected and boiled at 90 °C. Samples for PAS staining were prepared under reducing condition and a sample of methacholine-evoked saliva from a normal rat was added as control. The samples were then electrophoresed on precast 4–12% SDS-PAGE gel (NUPAGE Novex Bis-Tris gel, Invitrogen, Paisley, UK) according to the manufacturer's protocol. For the purpose of the immunoblotting equal amounts of protein from each experimental tissue was assessed with the NanoDrop ND-1000 Spectrophotometer (NanoDrop Technologies) (260/280 nm) and loaded on the gel.

Detection of glycoproteins was assessed via Periodic Acid Schiff staining. The gel was fixed in methanol and acetic acid, incubated with 1% periodic acid (for 15 min), rinsed with double distilled water and stain with Schiff's reagent.

For the immunoblotting the protein bands on the gel were electro-transferred to a nitrocellulose membrane for 1 h according to standard protocol (Invitrogen, UK, Paisley). The membrane was then blocked in a solution of milk powder and Tween-20 for 1 h (ProtoBlock kit, National Diagnostic). Rabbit polyclonal anti-ZZ3 delta (SMG B1/B2), was diluted (1:2000) in ProtoBlock and incubated with the membrane for 1 h. The membrane was then incubated with a secondary anti-rabbit HRP antibody (DAKO, Denmark) in a 1:2000 dilution for 1 h. After the primary and secondary antibody incubation the membrane was washed in TBS-T (20 mM TRIS, 150 mM NaCl, 0.1% Tween-20, pH 7.6) three times (5 min each). Bound antibody was visualized on photographic film following incubation with a chemiluminescent substrate containing 90 mM Coumaric acid (Sigma, UK) and 250 mM Luminol (Sigma, UK) in presence of hydrogen peroxide.

### 2.7 RNA extraction and real time PCR

Approximately 30 mg of frozen tissue was homogenized in 600 μl of RNA-Bee with a glass tissue grinder (Wheaton Science Products, NJ, USA). ARNeasy Mini Kit (Quiagen) was used for total RNA extraction according to manufacturer's protocol. RNA concentration was measured with the NanoDrop ND-1000 Spectrophotometer (*A*_260_/*A*_280_ ratio and *A*_260_/*A*_230_ ratio), and the integrity was assessed using the RNA 6000 Nano LabChip kit using the Agilent 2100 Bioanalyzer (Agilent Technologies).

0.5 μg of RNA was reverse transcribed with 200U M-MLV Reverse Transcriptase (Promega, WI, USA) for 55 min at 55 °C followed by 15 min at 8 °C using Random Primers (Promega, WI, USA) in a volume of 25 μl.

Real Time PCR was performed using the SensiMix Plus SYBR (Quantace) with the Corbett RotorGene 6000 system (Corbett Life Science) in a final volume of 25 μl. Relative gene quantification (expressed as fold change compared either to the normal or atrophic gland) was carried out using the Pfaffl equation which takes into account the PCR efficiency (Pfaffl, 2001). The data for the genes of interest (*smg-b* and *psp*) were normalized against *ubc* which has been shown to be a suitable housekeeping gene in our experimental conditions (Silver et al., 2008). The following primers were used in the reaction:

*smgb* Fw 5′GGACGTGGAGTCAGTTTGGT 3′, Rev 5′TTCATCACCATTGGGAGACA 3′*psp* Fw 5′CCTTCTCCAAACVAAACCAA 3′, Rev 5′GTTGTGGCTTGCTGAAGT 3′ *ubc* Fw 5′AAGAGCCCTTCTTGTGCTTG 3′, Rev 5′GCAAGAACTTTATTCAAAGTGCAA-3′.

### 2.8 Statistical analysis

Results were expressed as means ±SEM (Standard Error of the Mean), and were statistically compared by paired Student’s *t*-test; *p*<0.05 was considered statistically significant.

## 3 Results

### 3.1 Gland weights

Following 2 weeks of ligation, submandibular glands showed a reduction in weights of more than 50% compared to the unoperated control (Table 1). In all the early deligation time points considered (3, 5 and 7 days) in this study, submandibular glands showed a significant increase in weight between 17% and 20% above the ligated glands. However after 5 and 7 days of deligation glands did not show a significant variation in weight compared with the 3 days. Overall the mean weight of submandibular glands during the early deligation (3, 5 and 7 days) was almost half the mean weight of normal control glands (Table 1).

### 3.2 Parenchymal elements

#### 3.2.1 Acinar cells

**Fig. 1:**
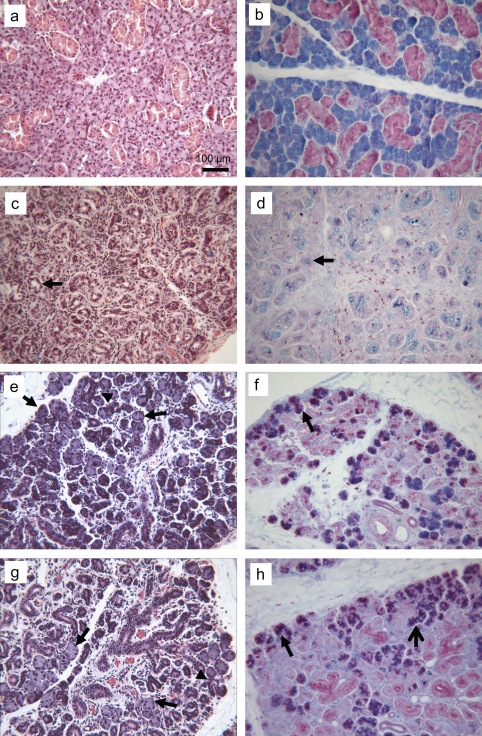
Histological comparison of normal gland, ligated (atrophic) gland and 5 or 7 days deligated glands. a, b Normal, unoperated gland stained with H & E and AB/PAS, respectively, showing the typical appearance of acinar and ductal cells. c, d H & E and AB/PAS-stained sections of 2 weeks ligated gland showed extensive inflammation, dilatation of the duct lumen (*arrow in* c), and loss of glycoproteins from the acini (*arrow in* d). e, g H & E staining of 5 and 7 days deligated glands respectively. Several acini and ductal cells have recovered some of their size (*arrows*). Branched structures were often visible (*arrowheads*). f, h AB/PAS staining of 5 and 7 days deligated glands respectively, showing the recovery of glycoproteins content in the acini (*arrows*) and in the granular ducts (*open arrow*).

**Fig. 2:**
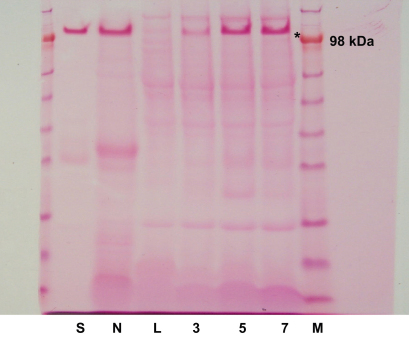
PAS staining of glycoproteins resolved by SDS-PAGE gel for: Methacholine-evoked saliva from normal submandibular gland (Lane S) and submandibular gland homogenate from unoperated (normal) submandibular gland (Lane N); 2 weeks ligated (atrophic) gland (Lane L); 3 days deligated gland (Lane 3); 5 days deligated gland (Lane 5) and 7 days deligated gland (Lane 7). Molecular weight marker (M). The asterisk marks the rat low molecular mucin (∼114 KDa).

**Fig. 3:**
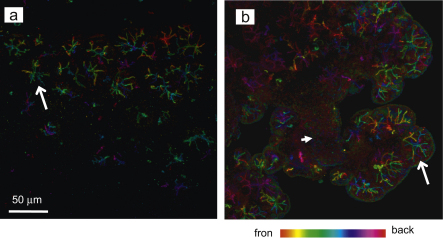
AQP5 expression in the (a) 5 and (b) 7 days regenerated submandibular gland. Collagenase-digested cells were incubated with an anti-AQP5 antibody. Different colours represent differences in depth of field. Aquaporin 5 is expressed in the acinar cells (arrow) whilst the ducts are negative (small arrow).

Histological examination (H&E) of the 3, 5 and 7 days deligation time points showed the presence of several normal-looking acinar cells on both the edge of the lobules and in the middle of the parenchyma (Fig. 1e and g). These normal-looking acinar cells, which were not apparent in the atrophic tissue (Fig. 1c and d), exhibited normal morphology (Fig. 1: comparison between a,b and e,f,g,h). AB/PAS histochemistry revealed that most acinar cells at 5 and 7 days of deligation had recovered their glycoprotein content, which was lost during atrophy (Fig. 1d, f, and h). Accordingly a recovery in the expression of the rat low molecular mucin (114 kDa), normally secreted in saliva (Fig. 2 lane S), was identified in the deligated glands in a PAS-stained gel, based on apparent molecular weight (Fig. 2 lane N,L,3,5 and 7). In a previous paper by the authors (Cotroneo et al., 2008) the expression of the water channel aquaporin 5 (APQ5) was shown to decrease dramatically during atrophy and to re-appear in some acini during the early stages of glandular regeneration (3 days). In the current study immunocytochemistry at 5 and 7 days of deligation, revealed increased expression of APQ5 in the majority of the acinar cells (Fig. 3).

#### 3.2.2 Ducts

One of the main morphological features of atrophic glands was an increase in the proportion of ducts (Fig. 1c). This characteristic was still notable in the 5 and 7 days deligated glands, however the lumen of the duct was smaller than in the atrophic gland suggesting a recovery of duct cells cytoplasm (Fig. 1e and g). Furthermore, at these stages of deligation the granular ducts started to recover their granule content (Fig. 1h) as noted previously (Tamarin, 1971a, 1971b, 1971c).

#### 3.2.3 Characterization of the branched structures

**Fig. 4:**
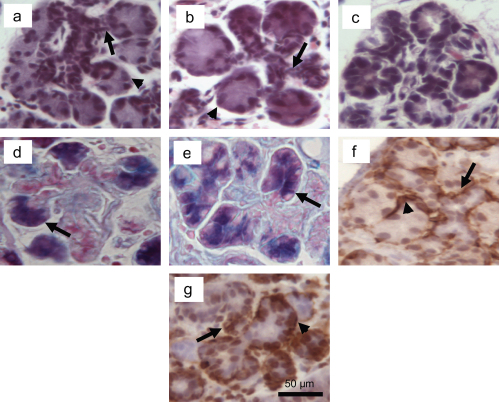
Morphological features of the branched structures. a, b H & E staining of the branched structures in respectively 5 and 7 days deligated glands, characterized by short ducts (*arrows*) ending with acini (*arrowheads*). c Typical embryonic (e20) branched structures. d, e AB/PAS of 5 and 7 days deligated gland respectively, showing presence of glycoprotein in the acini at the end of the branching (*arrows*). Smooth muscle actin immunohistochemistry of 5 (f) and 7 days (g) deligated showing presence of myoepithelial cells around the acini (*arrowheads*) and the acinar-duct junction (*arrows*); sections were counterstained with haematoxylin. Fig. g shows background staining in few nuclei.

**Fig. 5:**
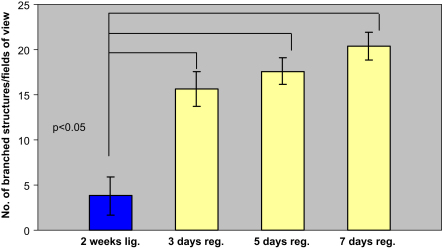
Mean number (±SEM) of branched structures per field of view (×200 magnification) in the 2 weeks ligated (*2 weeks lig.*) and the 2 weeks ligated plus 3, 5 or 7 days deligated (*3 days reg.; 5 days reg.; 7 days reg.*) submandibular gland stained by H&E. The number of the branched structures increased at 3 days of regeneration compared to the 2 weeks ligated gland (*p*<0.05, 5 observation from 5 rats, *n*=25), however it did not increase further (*p*>0.05) during the progression of the regeneration.

In both the 5 and 7 days deligated glands, H&E staining revealed the presence of peculiar branched structures characterized by at least three short ducts ending with mature or immature acini (Fig. 4a and b). These branched structures are absent in the normal gland (Fig. 1a), and interestingly they displayed a configuration very similar to the structures occurring in the embryonic submandibular gland during the branching morphogenesis (Fig. 4c, for purpose of comparison). These structures were more frequent in the 3 days deligated (15.6±1.9 *p*<0.5) glands compared to the atrophic (3.8±2.1); however during the progression of the deligation they did not significantly change in number (Fig. 5). At both the deligation time points several acini at the end of the branched structures were also stained with AB/PAS revealing the presence of glycoproteins (Fig. 4d and e).

Smooth muscle actin immunostaining revealed the presence of myoepithelial cells surrounding the acini of the branched structures along with the acinar-duct junction (Fig. 4f and g).

### 3.3 Cell proliferation

**Fig. 6:**
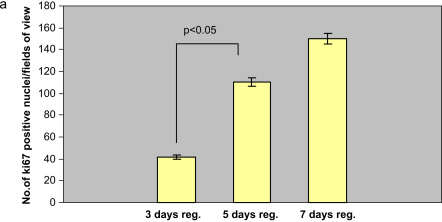

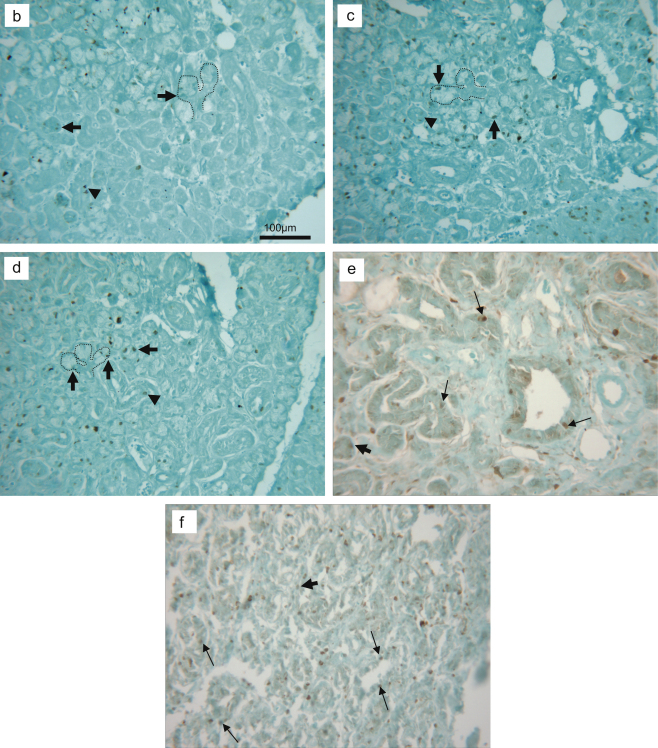
Cell proliferation in the deligated glands. a, number of ki-67 positive nuclei per field of view (×200 magnification) in the 2 weeks ligated plus 3 or 5 or 7 days deligated (*3 days reg.; 5 days reg.; 7 days reg.*) submandibular gland. 5 days deligated gland showed an increase in the number of proliferating cells (*p*<0.05, 3 observation in 5 rats, *n*=15) compared to the 3 days. b, c, d ki-67 immunostaining (counterstained with Light Green dye) in the 2 weeks ligated plus 3 or 5 or 7 days deligated respectively, showing mostly proliferating acinar cells (*arrows*), also present at the end of the branched structures (*dashed silhouettes*), and occasionally ductal cells (*arrowheads*). e, f ki-67 immunostaining of normal adult and ligated rat submandibular gland respectively. Normal gland (e) showed proliferation of ductal cells (arrow) and occasionally acinar cells (*thick arrow*). The ligated gland (f) revealed proliferation of ductal cells (*arrows)* and some non-parenchymal cells (*thick arrow*). All tissue sections were counterstained with Light Green.

Although the weight of the glands did not change significantly across the deligation time points, we detected an increase in the number of proliferating cells between day 3 and day 5 (*p*<0.05), using ki-67 (Fig. 6a). The ki-67 immunohistochemistry in all the deligation time points showed that dividing nuclei are scattered across the sections and seemed to localize mostly in the acini and only occasionally in the ducts (Fig. 6b–d). We were able to detect proliferation in the acinar cells at the end of the branched structures (Fig. 6b–d dashed silhouette). The adult normal submandibular gland showed a low level of cellular proliferation within the ductal compartment and occasionally of acinar cells (Fig. 6e). The ligated gland was characterized by proliferation of several ductal cells and some non-parenchymal cells (Fig. 6f).

### 3.4 Perinatal proteins

#### 3.4.1 SMG-B expression and localization

**Fig. 7:**
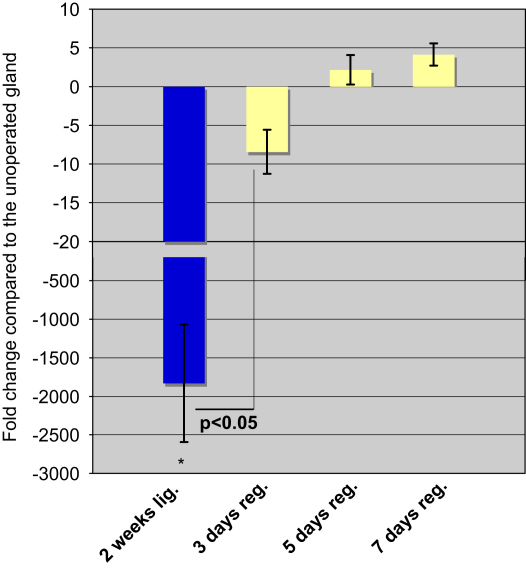
SMG-B mRNA expression level in the deligated glands. Real time PCR analysis showed an increase in *smg-b* expression in 3 days deligated gland (*3 days reg*.) compared to the atrophic gland (baseline) (^*^p<0.05). The level of SMG-B did not increase further during the progression of the regeneration (day 5 and 7). At all time points of regeneration the level of *smg-b* transcript was not up-regulated compared to the normal gland.

**Fig. 8:**
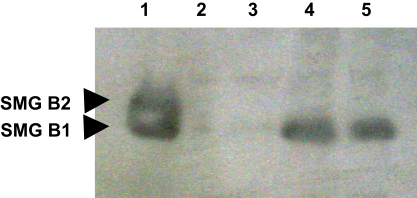
Western Blotting showing the expression of both SMG-B isoforms (B1 26 kDa and B2 27.5 kDa) in the unoperated (lane 1), 2 weeks ligated (lane 2), 3 days (lane 3), 5 days (lane 4) and 7 days deligated (lane 5) submandibular gland. In the normal adult gland both isoforms are present. After atrophy SMG-B1 isoform started to re-appear at 3 days and increased in the 5 and 7 days deligated glands.

**Fig. 9:**
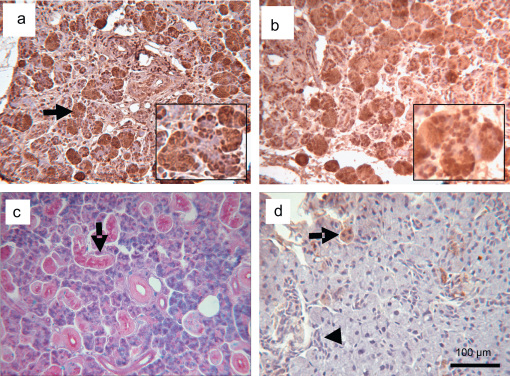
a, b SMG-B immunohistochemistry of 5 & 7 days and 8 weeks deligated submandibular glands respectively. In both the 5 & 7 day deligated glands SMG-B exclusively localized in the acinar cell (*arrows*) whilst the duct are negative. The acini at the end of the branched structures are also positive (*double arrows inset*). c AB/PAS staining of 8 weeks deligated glands showing recovery of the secretory granules in the acini and in the granular duct cells (*arrow*). d SMG-B immunohistochemistry 8 weeks deligated submandibular glands; most of the acini are now negative, except for few occasional cells (*arrow*). The intercalated ducts cells do not show immunoreactivity (*arrowhead*). All sections were counterstained with haematoxylin. The occasional nuclear staining is an artefact due to antigen retrieval pre-treatment.

We evaluated the mRNA expression level of *smg-b* in both the atrophic and the deligated glands, using real time PCR analysis. The atrophic glands when compared with the normal glands, revealed a dramatic reduction in the expression level of *smg-b* (∼1800 fold change, *p*<0.05). Interestingly when the deligated glands were compared with the atrophic it showed a significant increase in *smg-b* expression of more than 200 fold occurring at 3 days, (*p*<0.05) (Fig. 7). Although 5 and 7 days deligated glands showed a trend in the increase of *smg-b* expression, the difference in folds change between 3 and 5 days and between 5 and 7 days was not statistically significant. In none of the time points of regeneration was there up-regulation of *smg-b* compared to the normal gland. As with the mRNA level, the SMG-B protein was greatly reduced in the atrophic glands compared to the normal gland. By day 3 there was greater expression than the atrophic level which further increased in the 5 and 7 days deligated glands (Fig. 8 lane 2, 3, 4 and 5). The Western blotting also showed the presence of a double band in the adult gland corresponding to the two isoforms of SMG-B (B2 at 27.5 kDa and B1 at 26 kDa) (Fig. 8 lane 1). Interestingly only the band corresponding to SMG B1 seemed to be expressed in the deligated glands (Fig. 8 lane 3, 4 and 5). The anti SMG-B immunohistochemistry on the 5 and 7 days deligated glands showed a change in the localization of the protein from the normal gland (Man et al., 1995). During the deligation SMG-B was exclusively expressed in the acinar cell, including those at the end of the branched structures, whilst the ducts were negative (Fig. 9a and b). At 8 weeks of deligation when the glands had recovered most of their acini along with their secretory proteins (Fig. 9c) SMG-B was still not expressed in the ducts and progressively disappeared from the acini (Fig. 9d).

#### 3.4.2 SMG-A/PSP expression

**Fig. 10:**
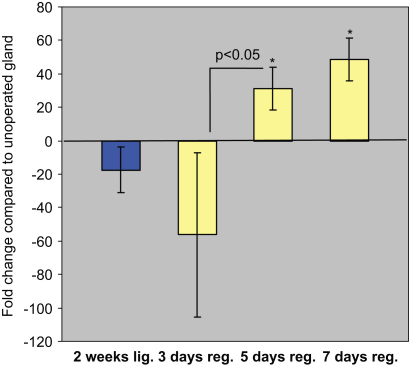
PSP mRNA expression level in the 2 weeks ligated (*2 weeks lig*.), 2 weeks ligated plus 3 or 5 or 7 days deligated (*3 days reg.; 5 days reg.; 7 days reg.*) using the unoperated adult submandibular glands as baseline. The 5 days deligated gland (*5 days reg*.) revealed an increase in SMG-B expression (*p*<0.05) compared to the 3 days deligated gland. The level of PSP did not increase further during the progression of the regeneration (day 7). The *psp* transcript was up-regulated at 5 and 7 days of regeneration compared to the normal gland (30 and 50 fold change respectively; ^*^p<0.05).

We evaluated the mRNA expression level of another perinatal protein, SMG-A/PSP, in both the atrophic and the deligated glands. Real time PCR analysis did not show any change in *psp* expression either in the atrophic gland compared to the normal or in the 3 days deligated gland compared to the atrophic. However we detected a significant increase in psp transcript level between the 3 and the 5 days deligated glands. The fold change difference between 5 and 7 days deligated glands was not statistically significant, however at both time points the *psp* level was significantly higher than the normal gland (Fig. 10).

## 4 Discussion

In this study the duct ligation-deligation technique was used to investigate the regeneration of secretory tissue in the rat submandibular gland. After removal of the obstruction the glands showed recovery in weight together with recovery of acinar secretory glycoproteins, such as mucin and functional proteins such as AQP5 along the apical membrane. All these features support the idea that deligation induces submandibular gland regeneration as previously described (Cotroneo et al., 2008; Osailan et al., 2006a, 2006b).

Following duct ligation previous studies have suggested that residual shrunken acini persist during atrophy (Cotroneo et al., 2008; Tamarin, 1971a, 1971b, 1971c, 1979). These acini are thought to be the first cell population to actively proliferate (day 2), followed later by differentiation and proliferation of newly formed acinar cell (day 4). Ductal cell proliferation also occurs during ligation, but to a much lesser extent, and rapidly decreases in early stages of regeneration (Takahashi et al., 2004a, 2004b). In the current study the early proliferation and recovery of the residual acini accounted for the regain of the rat low molecular weight mucin seen at 3 days.

In an earlier study on 3 day deligated submandibular gland, we have reported the presence of acinar-ductal branched structures characterized by at least three short ducts ending with immature acini which were significantly more frequent in the regenerated gland than in the atrophic (Cotroneo et al., 2008). Due to their similarity with the structures occurring during embryonic branching morphogenesis, we referred to them as embryonic-like branched structures and we proposed them as a source of newly differentiated acinar cells. In the current study on later regeneration time points (5 and 7 days), we have identified normal-looking acinar cells developing directly from these embryonic-like branched structures. This data further supports the previously suggested correlation between the formation of new secretory tissue during the regeneration and the early stage of glandular development (Cotroneo et al., 2008; Tamarin, 1971a, 1971b, 1971c, 1979). Although in the current study the number of these embryonic-like structures did not change during the progression of the regeneration, the ending acini appeared to undergo active mitosis. Moreover they appeared morphologically more differentiated in the 5 and 7 days than in the 3 days regenerated gland (Cotroneo et al., 2008). These regenerating acinar cells also expressed a general array of secretory glycoproteins including the rat low molecular mucin, which contributed further to the apparent increased AB/PAS staining seen at these time points (day 5 and 7).

Further proof that the terminal acinar cells were differentiating into mature acini was the presence of surrounding myoepithelial cells. During glandular development myoepithelial cell differentiation starts in the late embryonic stage and carries on postnatally in parallel with the maturation of acinar cell differentiation (Cutler and Chaudhry, 1973; Redman, 1994). Eventually in the adult submandibular gland they remain localized around the mature acini and the intercalated duct (Garrett and Parsons, 1973; Nagato et al., 1980). In our study the 5 and 7 days regenerated glands had myoepithelial cells around both the acini arising from the embryonic-like branched structures and the acinar-duct junction, reflecting the arrangement of the normal gland. Myoepithelial cells covering newly formed acini have been previously described during submandibular regeneration (Takahashi et al., 1997, 2004a, 2004b), raising the question of whether or not these cells may be play a role in supporting acini differentiation.

In order to further characterize the correlation between the glandular regeneration and the developmental stage, we investigated the molecular features of the newly formed acinar cells assessing the expression of the perinatal proteins SMG-B and SMG-A/PSP in the regenerated gland. The genes coding for SMG-A/PSP and SMGB share two similar domains at the 5′ and 3′ untranslated regions, respectively (Mirels and Ball, 1992). Although the function of these secretory proteins is still unknown, their three-dimensional conformation is shared with the BPI (bactericidial/permeability-increasing protein)/LBP (lipopolysaccharide-binding protein)/PLUNC (palate, lung and nasal epithelial clone) gene family, suggesting an antibacterial role (Wheeler et al., 2002).

In a previous study on an earlier time point of regeneration (3 days) we have reported the presence of a few immature acini localized on the edge of the lobules which exhibited a perinatal-like immunoreactivity for SMG-B (Cotroneo et al., 2008). In accordance with this previous result the current study shows the *smg-b* transcript to be significantly up-regulated at 3 days of deligation. As the regeneration progressed, at 5 and 7 days SMG-B was found expressed in the vast majority of the newly formed acini at the end of the branched structures on both the edge and the middle of the lobules, whilst the ducts were negative. The increased number of SMG-B positive acini between day 3 and day 5 was also reflected by increased amounts of protein, and suggests that this acinar cell subpopulation mostly account for the proliferation seen between day 3 and day 5. Although the expression of *smg-b* transcript was not significantly up-regulated at 5 days compared to the 3 days, it still showed a tendency to increase (*p*=0.07).

Following 8 weeks of regeneration the submandibular gland has been reported to recover its secretory function, suggesting among other things the presence of mature acini (Osailan et al., 2006a, 2006b; Carpenter et al., 2009). Interestingly in our study SMG-B expression was lost in the acini at 8 weeks of regeneration, indicating that functional, fully differentiated acinar cells no longer expressed this perinatal protein. The intercalated ductal cells, which in the normal adult submandibular gland still retain SMG-B expression (Man et al., 1995; Cotroneo et al., 2008), were negative at 8 weeks of regeneration. Although at this stage the gland is functional, ducts have not yet recovered their full function (Osailan et al., 2006a, 2006b). Therefore, it is possible that the expression of SMG-B may re-appear later once the ducts have fully recovered.

The localization of SMG-B in the new acinar cells originating from the branched structures, and its loss from the mature acini echoes the pathway of cytodifferentiation occurring during the perinatal stage. These findings suggest that a population of cell precursors, most likely the duct cells of the branched structures, have switched on the transient perinatal program of protein expression at some earlier stage of regeneration. This further suggests that the duct cells from which the new acinar cells originate, were pluripotent or at least undifferentiated cells. Whether these undifferentiated duct cells derive from de-differentiation of the ductal cells during the atrophy (Tamarin, 1971a, 1971b, 1971c; Okumura et al., 2003; Takahashi et al., 1998), or from a pre-existent population of pluripotent intercalated duct cells (Man et al., 1995; Kishi et al., 2006; Man et al., 2001) is still unclear. Nevertheless the up-regulation of SMG-B at 3 days could be the consequence of the commitment of these undifferentiated duct cells towards the pro-acinar cell lineage (Type III), which differentiate later into mature acinar cell.

In contrast with the protein expression in the normal adult gland in all the regenerated glands we only detected a band at 26 kDa corresponding to the SMG-B1 isoform. During the perinatal stage the expression of two glycosylated isoforms of SMG-B are temporally regulated, with SMG-B2 preceding the expression of SMG-B1 (at perinatal day 3). The expression of SMG-B1 in the developing gland matches with the initiation of mucin biosynthesis, and it has been suggested that the same glycosyl transferase may be involved in the modification of both (Mirels et al., 1998). Interestingly in our study the amount of SMG-B1 isoform increased at day 5, concomitantly with the apparent increase of the low molecular weight mucin. The previous observations, which temporally linked the post-translational modification of SMG B1 and mucin, in our study might explain the parallel increase of these two proteins as the acinar cell differentiation progresses.

Our Real Time PCR analysis has also identified the presence of the *psp* transcript in all the experimental conditions considered. Previous studies based on Northern and Western-bloting provided controversial evidence about the presence of *psp* mRNA and protein in the adult gland (Mirels et al., 1998; Moreira et al., 1991). By using Real Time PCR we believe we have increased the sensitivity of the method and have obtained a better estimate of *psp* mRNA level. The present study shows an up-regulation of *psp* mRNA occurring at 5 days, which is probably part of the perinatal program of cytodifferentiation that is taking place at this stage of regeneration. We were not able to assess the localization of this SMG-A/PSP due to the lack of a suitable antibody specific for this protein. However we speculate that, like SMG-B, SMG-A/PSP is expressed in the newly formed acinar cell; although further experiments are needed to confirm this hypothesis. In our study we detected a gap in the up-regulation of *smg-b* and *psp*, it is worth remembering that such a gap in expression was not reported to occur during the glandular development (Mirels et al., 1998). *Psp* level did not change significantly after 2 weeks of atrophy when compared to the normal gland, in opposite to *smg-b*. This data allows the possibility that the population of undifferentiating duct cells, from which the new acini eventually originate, might have already started the process of commitment in the early atrophy with the expression of *psp*.

In conclusion, we have provided evidence that new acinar cells differentiate from the ductal cells after they underwent branching in a similar fashion described for the embryonic glandular development. Furthermore we have shown an up-regulation during the regeneration of the pro-acinar cells markers SMG-B and SMG-A/PSP. The localization of SMG-B in the newly differentiating acinar cell arising from the duct branched structures suggests that differentiation of these cells during regeneration follows the perinatal pathway of cytodifferentiation.

## Figures and Tables

**Table 1:** Mean weight of 2 weeks ligated (*2 weeks lig.*), of 2 weeks ligated plus 3 or 5 or 7 days or 8 weeks deligated (*3 days reg.*; *5 days reg.; 7 days reg.; 8 weeks reg.*) and unoperated (*normal*) adult submandibular glands.

**Group**	**Gland weight±SEM**	**Gland weight (% of normal)**
normal	0.208±0.007	/
2 weeks lig.	0.084±0.002*	40
3 days reg.	0.106±0.002*	50
5 days reg.	0.102±0.004	49
7 days reg.	0.106±0.008	50
8 weeks reg.	0.184±0.006**	88

* *p*<0.05; *n*=5 in each group.

** *n*=3.
